# Supplementary material for: The Campylobacter concisus BisA protein plays a dual role: oxide-dependent anaerobic respiration and periplasmic methionine sulfoxide repair
Source: mBio. 2023 Aug 21;14(5):e01475-23. doi: 10.1128/mbio.01475-23 (PMC10653797; doi:10.1128/mbio.01475-23)
Supplement: Supplemental material — Tables S1-S5, Fig. S1-S4, and supplemental experimental procedures. [file mbio.01475-23-s0001.pdf]

SUPPLEMENTARY DATA FOR:

The *Campylobacter concisus* BisA protein plays a dual role: oxide-dependent anaerobic respiration and periplasmic methionine sulfoxide repair

Stéphane L. Benoit[1,2#] and Robert J. Maier[1,2#]

Department of Microbiology,[1]
and
Center for Metalloenzyme Studies,[2]
University of Georgia, Athens, Georgia, 30602

Running title: N/S-oxide respiration and MetO repair in *C. concisus*

[#]To whom correspondence should be addressed:

Dr. Benoit (stefbens@uga.edu) or Dr. Maier (rmaier@uga.edu)

SUPPLEMENTARY TABLES

Table S1- Distribution of putative periplasmic Mo/W-bisPGD N/S-oxide reductases in 23 *C. concisus* sequenced strains.

**General information on C. concisus sequenced strains**							**Putative N/S oxide Reductase (locus tag, protein size in amino-acids, aa)**					
**Strain**	**GS**	**Source**	**Health Status**	**Genome Accession #**	**Chrom. size (kbp)**	**Plasmids**	**BisA (855 aa)**	**BisA' (835 aa)**	**DmsA (763 aa)**	**TorA (813 aa)**	**TorZ (817 aa)**	**Total number**
**13826 (BAA-1457)[a]**	2	Enteric	Gastroenteritis	CP000792-	2.05	2	13826_1395 (**855 aa**)	13826_1396 (**835 aa**)	13826_2315 (**763 aa**)	13826_1119 (**813 aa**)	13826_0406 (**817 aa**)	**5**
**ATCC 33237[b]**	1	Oral	Gingivitis	CP012541	1.84	0	**Absent**	**Absent**	33237_0068 (**763 aa**)	33237_0015 (**813 aa**)	33237_1371 (**817 aa**)	**3**
**51562[c]**	1	Enteric	Gastroenteritis	ANNI00000000	1.84	0	51562_0033 (**855 aa**)	**Absent**	**Absent**	51562_1174 (**813 aa**)	**Absent**	**2**
**51561[c]**	2	Enteric	Healthy	ANNH00000000	1.99	0	ATCC51561_1984 (**855 aa**)	**Absent**	51561_0090 (**763 aa**)	51561_1115 (**813 aa**)	51561_1698 (**817 aa**)	**4**
**UNSW1[c]**	2	Enteric	Gastroenteritis	ANNF00000000	1.94	0	UNSW1_1769 (**855 aa**)	**Absent**	**Absent**	UNSW1_1004 (**813 aa**)	UNSW1_1618 (**817 aa**)	**3**
**UNSW2[c]**	2	Enteric	CD	ANNJ00000000	2.01	0	UNSW2_0602 (**855 aa**)	**Absent**	UNSW2_1644 (**763 aa**)	UNSW2_0545 (**813 aa**)	UNSW2_1803 (**817 aa**)	**4**
**UNSW3[c]**	2	Enteric	CD	ANNE00000000	1.91	0	UNSW3_1681(**855 aa**)	**Absent**	**Absent**	UNSW1_0922 (**813 aa**)	UNSW3_1298 (**817 aa**)	**3**
**UNSWCD[c]**	2	Enteric	CD	AENQ00000000	1.81	1	UNSWCD_1448 (**855 aa**)	**Absent**	**Absent**	UNSWCD_1483 (**812 aa**)	UNSWCD_634 (**817 aa**)	**3**
**UNSWCS[c]**	2	Enteric	Gastroenteritis	ANNG00000000	2.11	0	UNSWCS_1639 (**855 aa**)	**Absent**	**Absent**	UNSWCS_0609 (**813 aa**)	**Absent**	**2**
**P1CDO2[d]**	2	Oral	CD	CP060707	2.03	0	CVT00_05565 (**855 aa**)	CVT00_05570 (**835 aa**)	CVT00_09035 (**763 aa**)	CVT00_00500 (**813 aa**)	CVT00_02880 (**817 aa**)	**5**
**P1CDO3[d]**	2	Oral		CP049266	2.05	0	CVT01_05350 (**855 aa**)	CVT01_05355 (**835 aa**)	**Absent**	CVT01_00445 (**813 aa**)	CVT01_02700 (**817 aa**)	**4**
**P2CDO4[d]**	2	Oral	CD	CP021642-	1.98	1	CCS77_1098 (**855 aa**)	**Absent**	CCS77_1749 (**763 aa**)	CCS77_0118 (**814 aa**)	CCS77_0577 (**816 aa**)	**4**
**P3UCB1[d,e]**	1	Enteric	UC	CP049238	1.83	0	G5B97_03290 (**855 aa**)	**Absent**	**Absent**	G5B97_00085 (**813 aa**)	G5B97_06745 (**817 aa**)	**3**
**P3UCO1[d,e]**	1	Oral	UC	CP049239	1.80	0	G5B98_03075 (**855 aa**)	**Absent**	**Absent**	G5B98_00085 (**813 aa**)	G5B98_06535 (**817 aa**)	**3**
**P10CDO-S2[d]**	1	Oral	CD	CP049274	1.93	0	**Absent**	**Absent**	**Absent**	CVT06_00080 (**813 aa**)	CVT06_07235 (**817 aa**)	**2**
**P11CDO-S1[d]**	2	Oral	CD	CP049264-CP049265	2.03	1	CVT07_05225 (**855 aa**)	CVT07_05230 (**835 aa**)	**Absent**	CVT07_00465 (**813 aa**)	CVT07_06815 (**817 aa**)	**4**
**P13UCO-S1[d]**	2	Oral	UC	CP060705	2.00	1	CVT08_04235 (**855 aa**)	**Absent**	**Absent**	CVT08_00450 (**813 aa**)	CVT08_02755 (**817 aa**)	**3**
**P15UCO-S2[d]**	2	Oral	UC	CP049234-CP049236	1.94	2	G5B99_03465 (**855 aa**)	**Absent**	**Absent**	G5B99_00065 (**813 aa**)	G5B99_07090 (**817 aa**)	**3**
**P26UCO-S2[d]**	1	Oral	UC	CP049270-CP049271	1.89	1	CVT15_03100 (**855 aa**)	**Absent**	**Absent**	CVT15_00085 (**813 aa**)	CVT15_06720 (**817 aa**)	**3**
**P27CDO-S2[d]**	1	Oral	CD	CP049272-CP049273	1.83	1	CVT17_03250 (**855 aa**)	**Absent**	**Absent**	CVT17_00085 (**813 aa**)	CVT17_06785 (**817 aa**)	**3**
**H9O-S2[d]**	2	Oral	Healthy	CP049232-CP049233	2.02	1	G5B96_04570 (**855 aa**)	G5B96_04565 (**835 aa**)	G5B96_08805 (**763 aa**)	G5B96_00575 (**813 aa**)	G5B96_07115 (**817 aa**)	**5**
**H1O1[d]**	1	Oral	Healthy	CP049237	1.86	0	**Absent**	**Absent**	**Absent**	G5B95_00085 (**813 aa**)	G5B95_06680 (**817 aa**)	**2**
**H16O-S1[d]**	2	Oral	Healthy	CP049263	1.98	0	CVS89_03655 (**855 aa**)	**Absent**	**Absent**	CVS89_00065 (**813 aa**)	CVS89_07280 (**817 aa**)	**3**
												**Average: 3.3**

Abreviations: GS, genomospecies; CD: Crohn's disease; UC, ulcerative colitis; Chrom, chromosome.

[a] Complete genome sequence of *C. concisus* 13826 (BAA-1457) available at https://www.ncbi.nlm.nih.gov/nuccore/NC_009802.2

[b] Complete genome sequence of *C. concisus* 33237 from Cornelius *et al.* (2017). Genome Announcements, 5 (29) e00711-17

[c] Draft genome sequence of *C. concisus* 51561, 51562, UNSW1, UNSW2, UNSW3, UNSWCD and UNSWCS from Deshpande *et al.* (2013). BMC Genomics, 14:585. http://www.biomedcentral.com/1471-2164/14/585.

[d] Complete genome sequence of *C. concisus* P1CDO2, P1CDO3, P2CDO4,…, H9O-S2, H1O1 and H16O-S1 from Liu *et al.* (2020). Microbial Genomics 2020; 6. DOI 10.199/mgen.0.000457.

[e] These two *C. concisus* strains were isolated from the intestine (P3UCB1) and the oral cavity (PU3CO1) of the same individual (patient with UC). Liu *et al.* (2020). Microbial Genomics 2020; 6. DOI 10.199/mgen.0.000457.

Table S2. Putative N- or S-oxide Reductases (N/SORs) found in *Campylobacter concisus* strains 13826, 33237 and 51562, and homologs in *Campylobacter jejuni* strains NCTC 11168 and 81-176

N/SOR Complex	Subunit name[a] (Cell localization)	Subunit Features	*Campylobacter concisus* strain			*Campylobacter jejuni* strain	
			(13826)[b]	(33237)[c]	(51562)[d]	(NCTC 11168)[e]	(81-176)[f]
N/SOR Complex I	**BisA (periplasmic)**	**Gene locus tag** (alternate tag)	**1395** (RS05585)	**not present**	**0033**	**0264c** (***torA***)	**0291** (***torA***)
		Proposed function and size (aa)	catalytic (855 aa)		catalytic (855 aa)	catalytic (838 aa)	catalytic (838 aa)
		Other features	Mo/W-*bis* PGD, [4Fe-4S],TAT		Mo/W-*bis* PGD, [4Fe-4S],TAT	Mo/W-*bis* PGD, [4Fe-4S],TAT	Mo/W-*bis* PGD, [4Fe-4S],TAT
		% identity/similarity to *Cc* 13826	100%		93/95%	65/78%	65/78%
	**BisA' (periplasmic)**	**Gene locus tag** (alternate tag)	**1396** (RS05590)	**not present**	**not present**	**not present**	**not present**
		Proposed function and size (aa)	catalytic? e^- transfer? (835 aa)				
		Other features	Mo/W-*bis* PGD, [4Fe-4S],TAT				
		% identity/similarity to *Cc* 13826	62/75% to BisA				
	**BisB (periplasmic)**	**Gene locus tag** (alternate tag)	**1398** (RS05595)	**not present**	**0245**	**0265c** (***torB***)	**0292** (***torB***)
		Proposed function and size (aa)	Cytochrome c (191 aa)		Cytochrome *c* (191 aa)	Cytochrome *c* (191 aa)	Cytochrome *c* (191 aa)
		Other features	Monoheme		Monoheme	Monoheme	Monoheme
		% identity/similarity to *Cc* 13826	100%		96/97%	43/60%	42/61%
	**Known or proposed substrate specificity**		**DMSO, MetO, BSO, NANO, TMAO**		**DMSO, MetO, BSO, NANO, TMAO**	**DMSO, TMAO**	**DMSO, TMAO**
N/SOR Complex II	**DmsA (periplasmic)**	**Gene locus tag** (alternate tag)	**2315** (RS09200)	**0068** (RS00345)	**not present**	**not present**	**1570** (Cju34) (***dmsA***)
		Proposed function and size (aa)	catalytic (763 aa)	catalytic (763 aa)			catalytic (774 aa)
		Other features	Mo/W-*bis* PGD, [4Fe-4S],TAT	Mo/W-*bis* PGD, [4Fe-4S],TAT			Mo/W-*bis* PGD, [4Fe-4S],TAT
		% identity/similarity to *Cc* 13826	100%	99/99%			27/42%
	**DmsB (periplasmic)**	**Gene locus tag** (alternate tag)	**2316** (RS09205)	**0069** (RS00350)	**not present**	**not present**	**1571** (Cju35) (***dmsB***)
		Proposed function and size (aa)	e^- transfer (183 aa)	e^- transfer (183 aa)			e^- transfer (218 aa)
		Other features	[4Fe-4S]	[4Fe-4S]			[4Fe-4S]
		% identity/similarity to *Cc* 13826	100%	100%			39/54%
	**DmsC/NrfD (integral membrane)**	**Gene locus tag** (alternate tag)	**2318** (RS09215)	**0071** (RS00360)	**not present**	**not present**	**1572** (Cju36) (***dmsC***)
		Proposed function and size (aa)	MAP (294 aa)	MAP (294 aa)			MAP (288 aa)
		Other features	NrfD like, 8 TM	NrfD like, 8 TM			NrfD like, 8 TM
		% identity/similarity to *Cc* 13826	100%	83/91%			No significant similarity found
	**Known or proposed substrate specificity**		**Unknown**	**Unknown**			**Unknown**
N/SOR Complex III	**TorA (periplasmic)**	**Gene locus tag** (alternate tag)	**1119** (RS00445)	**0015**	**1174**	**not present**	**not present**
		Proposed function and size (aa)	catalytic (813 aa)	catalytic (813 aa)	catalytic (813 aa)		
		Other features	Mo/W-*bis* PGD, [4Fe-4S],TAT	Mo/W-*bis* PGD, [4Fe-4S],TAT	Mo/W-*bis* PGD, [4Fe-4S],TAT		
		% identity/similarity to *Cc* 13826	100%	94/97%	95/97%		
	**TorC/Y (periplasmic)**	**Gene locus tag** (alternate tag)	**1120** (RS00450)	**0014**	**1109**	**not present**	**not present**
		Proposed function and size (aa)	Cytochrome *c* (189)	Cytochrome *c* (189)	Cytochrome *c* (189)		
		Other features	Monoheme	Monoheme	Monoheme		
		% identity/similarity to *Cc* 13826	100%	94/98%	92/97%		
	**Known or proposed substrate specificity**		**Unknown**	**Unknown**	**Unknown**		
N/SOR Complex IV	**TorZ (periplasmic ?)**	**Gene locus tag** (alternate tag)	**0406** (RS07110)	**1371**	**not present**	**not present**	**not present**
		Proposed function and size (aa)	catalytic (817 aa)	catalytic (817 aa)			
		Other features	Mo/W-*bis* PGD, [4Fe-4S],TAT	Mo/W-*bis* PGD, [4Fe-4S],TAT			
		% identity/similarity to *Cc* 13826	100%	55/70%			
	**NapC/NirT (periplasmic, membrane-bound)**	**Gene locus tag** (alternate tag)	**1831** (RS00100)	**582 (possible)**	**not present**	**not present**	**not present**
		Proposed function and size (aa)	e^- transfer, MAP (234)	e^- transfer (190)			
		Other features	Tetraheme cyt *c*	Tetraheme cyt *c*			
		% identity/similarity to *Cc* 13826	100%				
	**Known or proposed substrate specificity**		**Unknown**	**Unknown**			

Abreviations: Mo/W-bis PGD, molybdenum or tungtsen *bis*-Pterin Guanine Dinucleotide; [4Fe-4S]: four iron-four sulfur cluster; MAP: membrane anchor protein; TAT: Twin Arginine Translocation signal sequence; TM: transmembrane
BSO: biotin sulfoxide; DMSO: dimethyl sulfoxide; MetSO: methionine sulfoxide; NANO: nicotinamide N-oxide; TMAO, trimethylamine-N-oxide

[a] Subunit name based on NCBI/Uniprot annotation or literature.

[b] Complete genome sequence of *C. concisus* 13826 (BAA-1457) available at https://www.ncbi.nlm.nih.gov/nuccore/NC_009802.2

[c] Complete genome sequence of *C. concisus* 33237 from Cornelius *et al.* (2017). Genome Announcements, 5 (29) e00711-17

[d] Draft genome sequence of *C. concisus* 51562 from Deshpande *et al.* (2013). BMC Genomics, 14:585.

[e] Complete genome sequence of C. jejuni NCTC11168 from Parkhill *et al.* (2000). Nature, 403:665-668.

[f] Complete genome sequence of C. jejuni 81-176 from Hofreuter *et al.* (2006). Infection and Immunity, 74:4694-4707.

Table S3. Percentage of identity/ similarity between five periplasmic Mo/W-bisPGD-containing N/SOR catalytic subunits

	BisA	BisA’	DmsA	TorA	TorZ
**BisA**		62/75	25/42	40/55	38/55
**BisA’**	62/75		22/39	39/56	39/57
**DmsA**	25/42	22/39		24/40	24/39
**TorA**	40/55	39/56	24/40		53/70
**TorZ**	38/55	39/57	24/39	53/70	

Table S4. Strains and plasmids used in this study.

Strain or plasmid	Relevant characteristics- Genomospecies	Source or reference
**Strain**		
C. concisus		
13826 (BAA-1457)	Parental strain (GS2). Isolated from human feces (gastroenteritis).	ATCC
33237	Parental strain (GS1). Isolated from gingival sulcus.	ATCC (1)
51562	Parental strain (GS1). Isolated from human feces (gastroenteritis).	ATCC (2)
E. coli		
TOP10	Cloning strain	Invitrogen
BL21 RIL	BL21 DE3 derivative. Host for protein overproduction; Cm^r	Novagen
**Plasmid**		
pUC20-cat	Source of *cat* cassette; Cm^r	(3)
pET21b	Expression vector; Amp^r	Invitrogen
pET-CcBisA	pET21b with *C. concisus* 13826_1395 gene cloned at *Nde*I-*Xho*I, native version.	This study
pET-CcBisA(His6)	pET21b with *C. concisus* 13826_1395 gene cloned at *Nde*I-*Xho*I, histidine-tagged version.	This study

References

1. Cornelius AJ, Miller WG, Lastovica AJ, On SLW, French NP, Vandenberg O, Biggs PJ. 2017. Complete genome Sequence of *Campylobacter concisus* ATCC 33237T and draft genome sequences for an additional eight well-characterized *C. concisus* Strains. Genome Announc 5:e00711-17.
2. Vandamme P, Falsen E, Pot B, Hoste B, Kersters K, De Ley J. 1989. Identification of EF group 22 campylobacters from gastroenteritis cases as *Campylobacter concisus*. J Clin Microbiol 27:1775-81.
3. Wang Y, Taylor DE. 1990. Chloramphenicol resistance in *Campylobacter coli:* nucleotide sequence, expression, and cloning vector construction. Gene 94:23-8.

Table S5. Primers used in this study.

Name	Sequence (5'-3')*	Usage
N/SOR Complex I		
bisA-1	ATGAACGAGAACAGACGAG	Construction of 13826_*1395* and 51562_*0033* mutants.
bisA-2	atccacttttcaatctatatcGGTTGCTTTTTGTAGCCAGC	Construction of 13826_*1395* and 51562_*0033* mutants
bisA-3	cccagtttgtcgcactgataaGAGTGGCTAGGCATGAAAG	Construction of 13826_*1395* and 51562_*0033* mutants
bisA-4	GATATTTACAAGAGCTGTGTG	Construction of 13826_*1395* and 51562_*0033* mutants
bisA-5	acggcc**catatg**AACGAGAACAGACGAG	Expression of 13826_1395 in *E. coli* (START codon)
bisA-6	gcgaca**ctcgag**ATCACTCCCCTGCTTTG	Expression of 13826_1395 in *E. coli* (brings STOP codon; native version)
bisA-7	gcgaca**ctcgag**TTTAGAAAATTTTGGCTC	Expression of 13826_1395 in *E. coli* (no STOP codon; histidine-tagged version)
bisB-1	CCATTTCTAGGCGGTTGCGC	Construction of 13826_*1396* mutant
bisB-2	atccacttttcaatctatatcGCCCTGCACCTGTCGAGTAG	Construction of 13826_*1396* mutant
bisB-3	cccagtttgtcgcactgataaCTTGGTACGCCAAGTGGTC	Construction of 13826_*1396* mutant
bisB-4	CTCTTTAAAGGCGCTAAGCT	Construction of 13826_*136* mutant
N/SOR Complex II		
dmsA-1	GGAGGCACCTATTGGCTGG	Construction of 13826_*2315* mutant
dmsA-2	atccacttttcaatctatatcGCTAGAGCTGCTCCGGTGCC	Construction of 13826_*2315* mutant
dmsA-3	cccagtttgtcgcactgataaCAAGACAACTCCAACTGGGG	Construction of 13826_*2315* mutant
dmsA-4	GCATACCAGCACTGTCGCTG	Construction of 13826_*2315* mutant
N/SOR Complex III		
torA-1	GATGGCAAGATGATCGG	Construction of 13826_*1119* and 51562_*1174* mutants
33-torA-1	ACACCAGTTG AAGTCGTCG	Construction of 33237_*0015* mutant
torA-2	atccacttttcaatctatatcTTTGAGTTGGTATTTGCCC	Construction of 13826_*1119*, 51562_*1174* and 33237_*0015* mutants
torA-3	cccagtttgtcgcactgataaACCATCCCATCTTCAAGATC	Construction of 13826_*1119*, 51562_*1174* and 33237_*0015* mutants
torA-4	CGTAGTTTCTGATTATTGAG	Construction of 13826_*1119*, 51562_*1174* and 33237_*0015* mutants

N/SOR Complex IV

torZ-1	AACACTATATCGGCAAATGG	Construction of 13826_*0406* mutant
torZ-2	atccacttttcaatctatatcAGGCTTGCTGCGCTTAAGGC	Construction of 13826_*0406* mutant
torZ-3	cccagtttgtcgcactgataaTAAGGGCACGCCAAAACGTG	Construction of 13826_*0406* mutant
torZ-4	GCGTCGAGCCTATGCTAACG	Construction of 13826_*0406* mutant
33-torZ-1	TTGCCAAGTA GCTCTTCTC	Construction of 33237_*1371* mutant
33-torZ-2	atccacttttcaatctatatcAGGCTAGCCGCGCCTAATGC	Construction of 33237_*1371* mutant
33-torZ-3	cccagtttgtcgcactgataaATGAGCCAGAGCAACACTGC	Construction of 33237_*1371* mutant
33-torZ-4	ATGCCGGACGGACATATGAAG	Construction of 33237_*1371* mutant

---

* Upper case letters indicate *C. concisus*-specific sequences; bold letters indicate newly generated restriction sites; *cat*-specific sequences are underlined. All primers were purchased from Integrated DNA Technology, Coralville, IA.

SUPPLEMENTARY FIGURES

Sup. Fig. S1. Multiple sequence alignment using Clustal Omega (https://www.ebi.ac.uk/Tools/msa/clustalo/)

Uniprot # → **Annotation**
A7ZFT3 → DmsA
A7ZDU4 → BisA
A7ZDU5 → BisA'
A7ZB52 → TorA
A7ZEP3 → TorZ

```
Conserved SYGW motif found in Rhodobacter sphaeroides DorA (associated with N/S-oxide reduction)
Tat-dependent signal peptide highlighted in yellow
Conserved Tat motif in bacteria xRRxFLK
tr|A7ZFT3|A7ZFT3_CAMC1      --MQRRSFLKGTGAALAAAGTAPSLFGMEQFEVDFNPKSYKNEENVEYHYLTCPRNCRDA  58
tr|A7ZDU4|A7ZDU4_CAMC1      MNENRRDFLKKGATAIAATPLLSGVTASNLFADEVKKGVVKNGETLTAAHW---------  51
tr|A7ZDU5|A7ZDU5_CAMC1      --MQRRKF-LKGSLALAATPFLGGCAFSG--NGSVKPSLVQNGSVKTSAYW---------  46
tr|A7ZB52|A7ZB52_CAMC1      --MKRRDFIKFSALAATAAQAS--------KIEGVTKTIFDQNKTFGANRF---------  41
tr|A7ZEP3|A7ZEP3_CAMC1      --MKRRDFLRLGALSAASLQAK--------ELDGAAQALFDKQSGLSANKF---------  41
                               :**.*    . : ::                      .: .

tr|A7ZFT3|A7ZFT3_CAMC1      CSMIAEIKDGKMVSIKGDPKHPLTQGTVCV--KGHTYAMHLYNADRIMHPMKRV------  110
tr|A7ZDU4|A7ZDU4_CAMC1      GMLKVTTKNGIAVKS--EP---IQKTSEIYNPLQHYTPDMI-YKSRIKHTMVRKSYLQNP  105
tr|A7ZDU5|A7ZDU5_CAMC1      GMMDVSVKDGVVTGS--KP---LSVLSSIPNPLRGYTADMI-YKCRIKHPMVRKSYLENP  100
tr|A7ZB52|A7ZB52_CAMC1      GLFWANTNSNQIVSV--DP---FEG-DKFPNTMNNSLPDLIQNESRVLYPYVRKSYLKAK  95
tr|A7ZEP3|A7ZEP3_CAMC1      GPFYVKTIAGRVVET--EP---FEG-DACPNELNNALLDHIQNESRVKFPFVRKSFLADP  95
                              : .    .  .    .*   :                 :    *: .   *
```

```
tr|A7ZFT3|A7ZFT3_CAMC1      -----GKKGEGKWEKISWDQALKEIAAKLTEIKEKFGGEALTEFVYS-GNEGHISKTI--  162
tr|A7ZDU4|A7ZDU4_CAMC1      DSPKPELRGIDEWVEVPYEEAIKLVAKELKKTRVQKGLQSVFAGSYGWKSSGNVHNSRIL  165
tr|A7ZDU5|A7ZDU5_CAMC1      DSPKPELRGQDEWVQVKYEDAIKLVARELKKTRAQRGLASVYAKSPAWKSSGNFNSSTTL  160
tr|A7ZB52|A7ZB52_CAMC1      GAAKSELRGKEEFVRVSWETALDLAAKALKENFDKYGPESIYGECYWWGGSGKISWGRTV  155
tr|A7ZEP3|A7ZEP3_CAMC1      NNPKPELRGKEEFVRVSWDEAIKLSAKILKENFDKYGSEAIYGQVYQWGSLGKVGHSQKT  155
                                   :*  :: .: :: *:.  *  *.:   : *  ::        . *:.

tr|A7ZFT3|A7ZFT3_CAMC1      ------APGNFFEKY----------------GATRLVRNPCDWPRYAGTPSVIGTDFSKD  200
tr|A7ZDU4|A7ZDU4_CAMC1      LHRFMNLSGGFVGSLGDYSTGASQIIMPHVVGSIEVYEQQTSWPVV--------------  211
tr|A7ZDU5|A7ZDU5_CAMC1      LARFMNLTGGFVGGLGDYSTGAGQVIMPHVMGGIEVYEQQTTWPVV--------------  206
tr|A7ZB52|A7ZB52_CAMC1      GHRMLKVLGGYVEESGDYSTGAGLVIMLHVLGNSAVYDAPTKWEAI--------------  201
tr|A7ZEP3|A7ZEP3_CAMC1      AKRLLNVLGGYVNELGGYSYGAATVIMPHITGSIDPTLAPTKWEAI--------------  201
                                    *.:.                  *          *

tr|A7ZFT3|A7ZFT3_CAMC1      ALEIDESDMYISWGSNEAYTA-VHWIRF-------AHRVKK----RGGKIIVINTIRIPL  248
tr|A7ZDU4|A7ZDU4_CAMC1      ---LENSKVVVIWGANPLATLRIAWTATDEQGFKYFEELKNKK---DIKVIVIDPIRSET  265
tr|A7ZDU5|A7ZDU5_CAMC1      ---LENSKVVVIWGSDPVATLRVGWTATDELAYKYLEDLKNS----DKEIIIIDPIKSLT  259
tr|A7ZB52|A7ZB52_CAMC1      ---AKNAKNVVFWGTDPLVTDQISWQPPTHDGYLGIKKIKE----AGIKTYSVCVFKNDT  254
tr|A7ZEP3|A7ZEP3_CAMC1      ---LKNAKTIVFWGTNPVVSNKIAIGVPLHNSYKYYDEIRKKGESGEMKIYSVDVYHNES  258
                                .::.  : **::   :  :              . :::       :   :   :

tr|A7ZFT3|A7ZFT3_CAMC1      ANQAD---MFIQLKPSSDPAFCLAVCKFLIEEDLYDHEFVKKYTTGFEDLVHECS-----  300
tr|A7ZDU4|A7ZDU4_CAMC1      AQYFD-KAQWIAPVPNTDTAMMLGMMHYLYESGKYDKEFIENYTYGFDKFLPYLLGKTDN  324
tr|A7ZDU5|A7ZDU5_CAMC1      GNFFDGKAKWIAPVPNTDTAMMLGMAYHLYKTKNYDKEFLESYTVGFDKFLPYLLGKKDG  319
tr|A7ZB52|A7ZB52_CAMC1      TRYLDS--EAIIVRPNTDVAMMLGMCHYLYENKLYDEEFIKKYTVGFNKFKDYLLGTTDK  312
tr|A7ZEP3|A7ZEP3_CAMC1      AKYFGA--KYLEVVPCTDTVMMIGMCNYLFAKGLYSKEFIEKYTVGFDKFKEYMLGTKDG  316
                             .  .     :   * :* .: :.:  .*     *..**::.** **:.:

tr|A7ZFT3|A7ZFT3_CAMC1      -LYTYGELSEMCGASVDQIKVFAREYAHAKAPAIMHGDGGQRHFNGARLVRAVTFLPVLT  359
tr|A7ZDU4|A7ZDU4_CAMC1      TPKNLEWASKICGIDKDTLKELADTFVSN-RTMLMSGWGMQRAHHGEQPHWAMVTLAAMI  383
tr|A7ZDU5|A7ZDU5_CAMC1      KPKTPKWASKICGVSEKVIKEFAVKMYKN-RTMIMGGWAMQRAHHGEQPYWMLVTLAAML  378
tr|A7ZB52|A7ZB52_CAMC1      VVKDINWASKICGVKAEDIAKFATALAKE-PSVIIAGRSLQRQDHGEMGFWGIVTLSAML  371
tr|A7ZEP3|A7ZEP3_CAMC1      VNKNLAWASKICGVSEQDLATFCEDLAKN-DSVIVSGYAIQRQDHGEMAYWALVTLSAML  375
                                    *::** . . :  :.          :: * . **  :*      :. * .:

tr|A7ZFT3|A7ZFT3_CAMC1      GALTKLGGGLFWAYVHVKGCFNFD--NCMPDLSPKD----KD-GKKIE------------  400
tr|A7ZDU4|A7ZDU4_CAMC1      GQIGLPGGGFGLSYHYSNGGAPTCKGAVIGGVNSASVGKFNEKGEFIGTDTGEFDKHGRF  443
tr|A7ZDU5|A7ZDU5_CAMC1      GQIGLPGGGFGFSYHYSGGGVPTSTATVVGGINPADIGVIKD-GKFKGYAKDVADE----  433
tr|A7ZB52|A7ZB52_CAMC1      GQIGKEGLGFEFNLYYSNG-ATDKIAPSLKGISTSISEKYDN------------------  412
tr|A7ZEP3|A7ZEP3_CAMC1      GHIGKEGCGFVTNDGMHKNADESFIAPKLAAFETKVPQKFID------------------  417
                            * :   * *:        .         :  ..        :

tr|A7ZFT3|A7ZFT3_CAMC1      --------------------------RQQVSYIEFGKGIQKENPTYLDKPINTVIKACIN  434
tr|A7ZDU4|A7ZDU4_CAMC1      VAKAAAVAGTGQSWLQKATNYAFPVARIADALLHPGKVIDHDGKKITYP----DIDFIYW  499
tr|A7ZDU5|A7ZDU5_CAMC1      ---------RLKAEIEEMQAASFPVARIADVLLNPGKTIDHNGKKITYP----DIDFIYW  480
tr|A7ZB52|A7ZB52_CAMC1      ---------VDGAPWKKFKNVTIPSSRSIEALQNPGKEIDYDGSKIKLP----HMRVAYM  459
tr|A7ZEP3|A7ZEP3_CAMC1      -----------SGLVPKTKGYELPNSRLIDALLSPGKEITRNGKSYKLP----KIRVMFN  462
                                                      *        ** *  :. .         :

tr|A7ZFT3|A7ZFT3_CAMC1      YNSNLMVTAPNTNLIKKRVMDDDFFLVVIDPYETDTCDYADYVLPGVTFMESEDIQN--D  492
tr|A7ZDU4|A7ZDU4_CAMC1      VGGNPLVHHQDTNTNL-KAWRKPRTVVVHEAYWTPTAKMADIVFPVTTEYERNDITMTGD  558
tr|A7ZDU5|A7ZDU5_CAMC1      AGGNPFGHQQNLNKLK-RAWQKPRTVVVNEIYWTPTAKMADIVFPVTTLYERDDITMVGD  539
tr|A7ZB52|A7ZB52_CAMC1      ASGSMFTRHQDVNNAV-KAWRKFDTVITAEPFWTSTAKLSDIVLPVALEVERNDINQSV-  517
tr|A7ZEP3|A7ZEP3_CAMC1      ANGSTFTRHPETNRAI-KAMQKVSAIITCEPFWTSTAKFSDIVLPAALECERTDIEIAN-  520
                             ... :    : *    :.  .    ::. : : * *.. :* *:* .    *  **
```

```
tr|A7ZFT3|A7ZFT3_CAMC1      QISGYVCYNAQSVKPLGEAKTNLEFFNALAKAMGYTEECFNWDSETV----CRRFLDTEF  548
tr|A7ZDU4|A7ZDU4_CAMC1      YSNMNIVPMKQVVEKYREAKDDYQIFTDLCKAYAKNLSIAYTDNGKDEFDWIKEYYDAAY  618
tr|A7ZDU5|A7ZDU5_CAMC1      YSNQYIMPMKQVVAKQNEAKDDYQIFSDLSKAYADGVAEIYTDGAKEPLDFIKSYYDSAA  599
tr|A7ZB52|A7ZB52_CAMC1      PTNEYIVAYKPVVEPMGESRSDYWICSQICKRWGREE--V-FTEGKDELGWAKEFYADAA  574
tr|A7ZEP3|A7ZEP3_CAMC1      STSEYLFAIKPLVAPFGESKSDFEIARLIAKEWGREE--A-FSEGKSELEWVKEIYEDAV  577
                              .  :      *    *:: :  :   :.*  .           .      :

tr|A7ZFT3|A7ZFT3_CAMC1      AKKQNI--TYEKLKSV--GWIKPFRTPETMKDQFPYYP----Y-APKDKLVFGTKSGKCE  599
tr|A7ZDU4|A7ZDU4_CAMC1      AQVKAVPELASDMKPFEEFWNENKPVTFASSQDSDNWVRLGEFREDPVLNALGTPSGLIE  678
tr|A7ZDU5|A7ZDU5_CAMC1      KVINENKALGVKMPKFSEWWEKNEPTKFDSTAENEAWVRHAEFREDPILNALGTPSGLIE  659
tr|A7ZB52|A7ZB52_CAMC1      EQAKGI--NV-KMPSFDEFWKEGYVRFEQDDEASRYYTRLSAFRENPHKNRLGTPSGKIE  631
tr|A7ZEP3|A7ZEP3_CAMC1      KKATGL--GYESMPSFDEFWQKGYFRFDKVDEKKRYFTNYKKFRDDPAANPLKTPSGKIE  635
                                       .:  .   * :              :     :        : * **  *

tr|A7ZFT3|A7ZFT3_CAMC1      LYSQTFKDAGYHPVIDLETDWDYYEKSNKFGKDYLKKYPLYFMTPGTQLQDNSNWGNMPY  659
tr|A7ZDU4|A7ZDU4_CAMC1      IYSDTIEKMGYDDCKAH---PTWFEPIEWLGMK-DKPAKYHMISAHPTDRLHSQLSQTSL  734
tr|A7ZDU5|A7ZDU5_CAMC1      IYSETIANMNYDDCKGH---AMWMEPTEWLGAK-KKSASIHLLNPHPSVRLHSQLGITSL  715
tr|A7ZB52|A7ZB52_CAMC1      LYSPTIAKFGYKDFAPH---VAWIEPFEWLGSEKAKKYPFSVTTPHSRYRLHSQLNNSII  688
tr|A7ZEP3|A7ZEP3_CAMC1      IYSGTVAGFGYDDCPPH---ATWLEPFEWLGAK-NKKYPIAISGAHSKFRLHSQLNNSVL  691
                            :** *.   .*.          : *  : :* .  *     .       : :*: .

tr|A7ZFT3|A7ZFT3_CAMC1      ILKRVIVKGNAELFMTREDMEARGIKNGDTVEATNEKGTAVFTAVETNQMNPGIVYAWNN  719
tr|A7ZDU4|A7ZDU4_CAMC1      R-EKYAIANREPVWINENDAKELGVKTGDLVCVFNARGEVLAGAYVTKNIKEGVVKLAEG  793
tr|A7ZDU5|A7ZDU5_CAMC1      R-DTYAVADREPILINTKNAKKLGIKTGDIVRVYNKRGEILAGAVVSDDVSYNVARLCEG  774
tr|A7ZB52|A7ZB52_CAMC1      R-NYAEVCAREPMLINVNDAKAKGIATGDVVRVFNDRGEILVGALVTDIIPEHVIAICEG  747
tr|A7ZEP3|A7ZEP3_CAMC1      R-HFNEIAEREPVLINPKTAEARGIKMGDVVKIYNDRGEILCGAFVTEDVPQNVVIVSEG  750
                              .   :  .  : :. :  :  *:  ** *   * :*  :  *  :. :   :    :.

tr|A7ZFT3|A7ZFT3_CAMC1      IWVKVTKSRTGANILCSDGVS----------DLGNGSTYTASFCEVKKAAKQEM------  763
tr|A7ZDU4|A7ZDU4_CAMC1      AWYDGF-----DSGICKNGSANVLTIDIPTSKLANGNISHTALVNIRKYQGDEAPKLTAF  848
tr|A7ZDU5|A7ZDU5_CAMC1      AWYDPD-----EKGVCKNGCINVLTNDIPASKLSNANISHTTLVNIKKFKGE-APELSAF  828
tr|A7ZB52|A7ZB52_CAMC1      AWYDPE--VLGEKSLCKHGCVNVLTRDKGTSSIAQSNCGHTILVNLEKYKGE-IKPITAF  804
tr|A7ZEP3|A7ZEP3_CAMC1      AWYDPD--VPGEKSLCLHGNLNVLTKDVPSSKMSQSNTAHTSLVEVEKFKGT-PKRVRAF  807
                             * .        . :* .*            .:.:..   : : ::.*

tr|A7ZFT3|A7ZFT3_CAMC1      ----------  763 DmsA
tr|A7ZDU4|A7ZDU4_CAMC1      SEPKFSK---  855 BisA
tr|A7ZDU5|A7ZDU5_CAMC1      KEPKFSV---  835 BisA′
tr|A7ZB52|A7ZB52_CAMC1      SKPKILQSL-  813 TorA
tr|A7ZEP3|A7ZEP3_CAMC1      DAPKIGARNA  817 TorZ
```

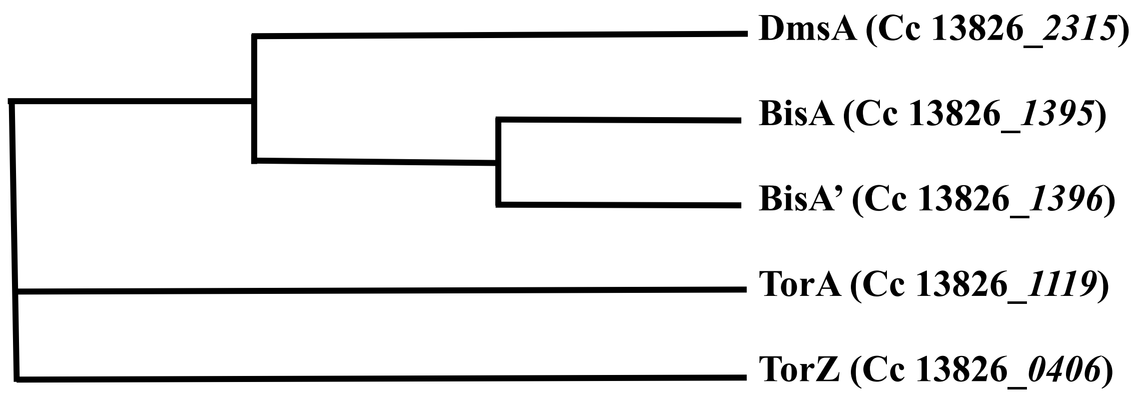

Sup. Fig. S2. Neighbor-joining phylogenetic tree without distance corrections.
Multiple sequence alignment and tree were made using Clustal Omega (https://www.ebi.ac.uk/Tools/msa/clustalo/)

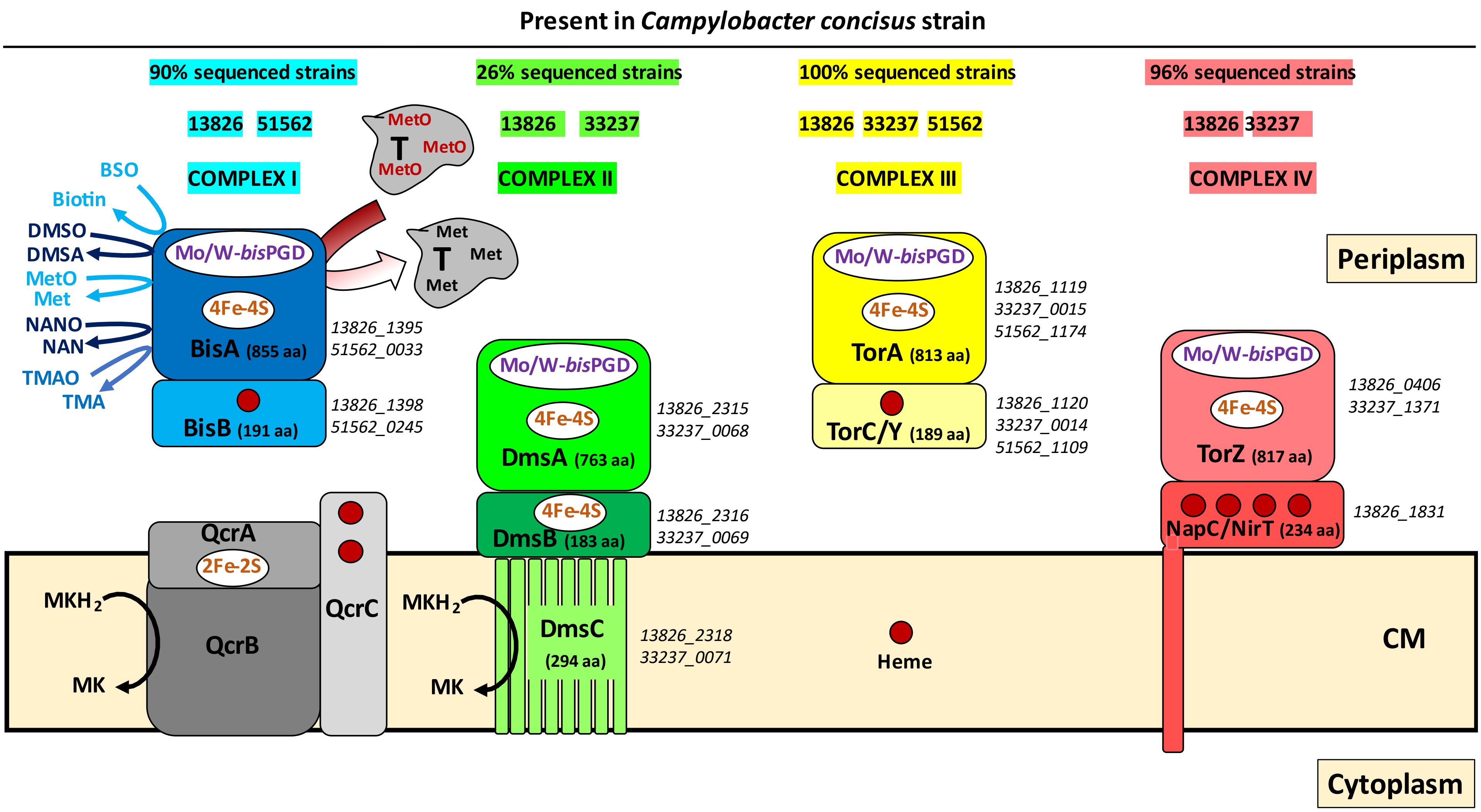

Sup. Fig. S3. Predicted cellular localization of several N- or S-oxide reductase complexes found in *C. concisus*, and predicted role(s) for BisA. Four putative N/SORs are shown here: BisA-B, DmsA-B-C, TorA-C/Y and TorZ-NapC/NirT. BisA', only found in some select *C. concisus* strains, is not depicted here. Note that the cytochrome associated with TorZ might be fully soluble (TorC/Y type). Electrons flow from menaquinones (MK) to the QcrABC complex and then to BisA-B, or possibly to TorA-C/Y or TorZ; alternatively, MK could give electrons directly to the DmsABC complex. BisA can repair MetO (to Met) in target proteins (shown here as "T").

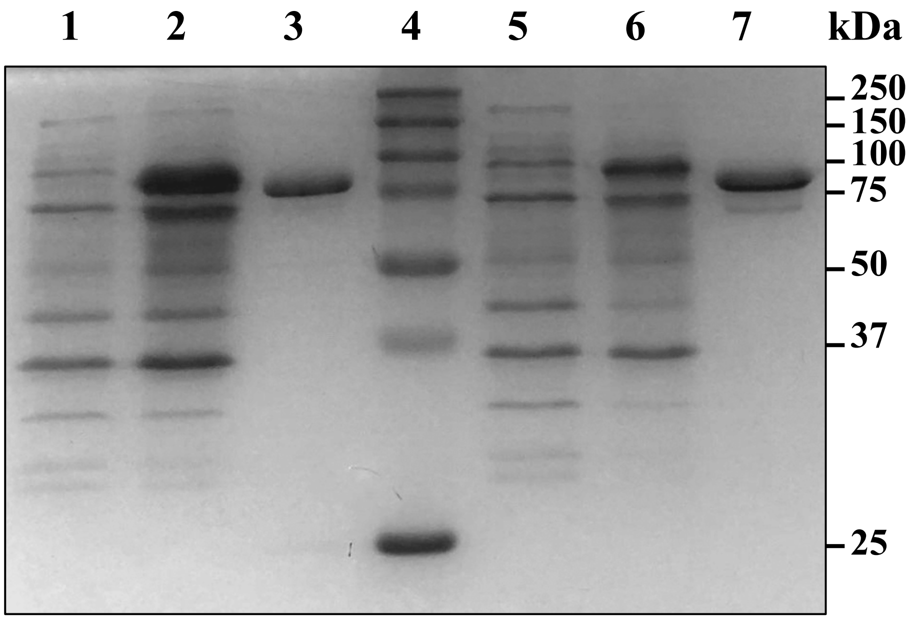

Sup. Fig S4. SDS-12.5% PAGE showing expression and purification of recombinant CcBisA and CcBisA-His$_6$.

Crude extract of uninduced (lane 1) and IPTG-induced (lane 2) *E. coli* RIL/pET-*Cc*BisA; FPLC-purified *Cc*BisA (lane 3);

Crude extract of uninduced (lane 5) and IPTG-induced (lane 6) *E. coli* RIL/pET-*Cc*BisA-His$_6$; Ni-NTA-purified *Cc*BisA-His$_6$ (lane 7).

Molecular mass standards are shown in lane 4, with mass in kDa indicated on the right.

The expected molecular mass is 92.19 kDa for mature *Cc*BisA (*i.e.*, without TAT-signal sequence), and 93.25 kDa for mature *Cc*BisA-His$_6$.

SUPPLEMENTARY MATERIALS AND METHODS

Detailed information on *C. concisus* mutant construction.

Construction of Δ*bisA* mutants in strains 13826 and 51562. The same set of four primers was used to construct Δ*bisA* mutants in both parental strains. Briefly, primers BisA-1 and BisA-2 were designed to amplify a 490 bp-long DNA sequence containing the beginning of the *bisA* ORF (13826_*1395*; 51562_*0033*), and part of the *cat* gene promoter. Primers BisA-3 and BisA-4 were designed to amplify a 400 bp-long DNA sequence containing the 3' end of *cat*, the 3' end of *bisA* and some sequence downstream of *bisA*. The final amplification step with both PCR products, the *cat* cassette and primers BisA-1 and BisA-4 generated a 1,600 bp-long product containing the Δ*bisA::cat* DNA sequence, which was then subsequently used to generate Δ*bisA* mutants in strains 13826 and 51562 (both strains possess *bisA*, whereas strain 33237 does not).

Construction of Δ*bisA'* mutant in strain 13826. Primers BisA'-1 and BisA'-2 were designed to amplify a 500 bp-long DNA sequence containing the beginning of the *BisA'* ORF (13826_*1396*), and part of the *cat* gene promoter. Primers BisA'-3 and BisA'-4 were designed to amplify a 540 bp-long DNA sequence containing the 3' end of *cat* and the 3' end of the *bisA'* gene. The final amplification step with both PCR products, the *cat* cassette, and primers BisA'-1 and BisA'-4 generated a 1740 bp-long PCR product containing the Δ*bisA'::cat* DNA sequence, which was then subsequently used to generate a Δ*bisA'* mutant in strain 13826 (*e.g.*, the only strain used in the study that contains *bisA'*).

Construction of Δ*dmsA* mutant in strain 13826. Primers dmsA-1 and dmsA-2 were designed to amplify a 560 bp-long DNA sequence containing some sequence upstream of, and the beginning of the *dmsA* ORF (13826_*2315*), and part of the *cat* gene promoter Primers dmsA-3 and dmsA-4 were designed to amplify a 820 bp-long DNA sequence containing the 3' end of *cat*, the 3' end of *dmsA* and some sequence downstream of *dmsA*. The final amplification step with both PCR products, the *cat* cassette, and primers dmsA-1 and dmsA-4 generated a 2,080 bp-long PCR product harboring the Δ*dmsA::cat* DNA sequence, which was then subsequently used to generate a Δ*dmsA* mutant in strain 13826 (*e.g.*, only strain used in the study haboring the *dmsABC* locus).

Construction of Δ*torA* mutants in strains 13826, 33237, and 51562. The same set of four primers was used to construct Δ*torA* mutants in the three parental strains, with the exception of 33237 Δ*torA* that required an additional sequence-specific primer. Primers torA-1 and torA-2, or 33-torA-1 and torA-2 were designed to amplify a 655 bp-long DNA sequence (or 640 bp-long for 33237) that contained some upstream flanking sequence and the beginning of the *torA* ORF (13826_*1119*; 33237_*0015*; 51562_1174), and part of the *cat* promoter. Primers torA-3 and torA-4 were designed to amplify a 820 bp-long DNA sequence containing the 3' end of *cat* and the 3' end of *torA*. The final amplification step with both PCR products, the *cat* cassette and primers torA-1 (or 33-torA1) and torA-4 generated a 2,160 bp-long (2145-bp for strain 33237) product containing the Δ*torA::cat* DNA sequence which was then subsequently used to generate Δ*torA* mutants in *C. concisus*.

Construction of Δ*torZ* mutants in strains 13826 and 33237. Due to high genetic diversity at the *torZ* locus between both strains, specific primers had to be designed for each strain. Primers torZ-1 and torZ-2 and primers 33-torZ-1 and 33-torZ-2 were designed to amplify a 745 bp-long (for strain 13826) and a 680 bp-long (for strain 33237) DNA sequence, respectively; both contained some sequence upstream of, and the beginning of the *torZ* ORF (13826_*0406*; 33237_*1371*), as well as the start of the *cat* promoter. Primers torZ-3 and torZ-4, and primers 33-torZ-3 and 33-torZ-4 were designed to amplify a 820 bp-long DNA sequence (for strain 13826) and a 600 bp-long DNA sequence (for strain 33237) respectively, containing the 3' end of *cat*, the 3' end of *torZ* and some sequence downstream of *torZ*. The final amplification step with both PCR products specific for each strain, the *cat* cassette and either primers torZ-1 and torZ-4, or primers 33-torZ-1 and 33-torZ-4, generated a 2,265 bp-long PCR product (for strain 13826) and a 1,980 bp-long PCR product (for strain 33237) respectively. Each construct was subsequently used to generate Δ*torZ* mutants in *C. concisus* strain 13826 and strain 51562, respectively.

Expression and purification of *Cc*BisA or *Cc*BisA(His)$_6$. *C. concisus* BisA (*Cc*BisA) was expressed in *E. coli* (BL21 RIL host strain) either as native recombinant (*Cc*BisA) or as hexahistidine-tagged protein (*Cc*BisA-His$_6$). Briefly, *C. concisus* 13826 genomic DNA was used as template in two independent PCR reactions. Primer bisA-5 (Table S5) was used with either primer bisA-6 (stop codon) or primer bisA-7 (no stop codon) to amplify a ~2,600 bp DNA sequence containing the whole *bisA* ORF (including the TAT signal sequence), as well as to

incorporate a 5' *Nde*I and a 3' *Xho*I restriction site, respectively. Both PCR products were digested with *Nde*I and *Xho*I, gel-purified and cloned into similarly digested pET21b plasmid, generating pET-CcBisA or pET-CcBisA-His$_6$, respectively. *E. coli* BL21 RIL strain harboring either plasmid was grown at 37°C in 800 mL LB supplemented with ampicillin (100 mg/L) and chloramphenicol (30 mg/L) to an OD_{600} of 0.5-0.7. Cultures were cooled for 20 min at 20°C, and protein expression was induced by four additions (20 µM every hour) of isopropyl β-D-1-thiogalactopyranoside (IPTG). Cells were harvested by centrifugation (15,000 x *g*, 20 min, 4°C) and subsequent steps were performed at 4°C. For purification of native recombinant *Cc*BisA, cells were washed with 200 ml of 20 mM Tris(hydroxymethyl)aminomethane (Tris)/HCl, pH 8.0 with 1 mM dithiothreitol (DTT) (buffer A), resuspended in 4 ml of the same buffer, and lysed by two passages through a cold French pressure cell at 20,000 lb/in^2. Unbroken cells and cell debris were removed by centrifugation at 15,000 x *g*, and the cell-free supernatant was subjected to ultracentrifugation at 80,000 x *g* for 1 h. The membrane-free supernatant was then applied to a 5-ml Q Sepharose anion exchange column (GE healthcare, Piscataway, NJ), using a ÄKTA-FPLC™ system. A linear gradient with buffer B (buffer A with 1 M NaCl) was used to purify the protein. Fractions of interest were pooled, concentrated to a final volume of 1 ml using a 10 kDa MWCO Amicon device (Millipore, Billerica, MA), before being loaded on a size exclusion column (Sephacryl S200, GE Healthcare) equilibrated with buffer C (equimolar mix of buffers A and B). Fractions containing *Cc*BisA were pooled and concentrated again. For purification of recombinant *Cc*BisA(His)$_6$ version, cells were washed with 200 ml of 50 mM NaH_2PO_4 (pH 8), containing 300 mM NaCl and 5 mM imidazole (buffer D) and resuspended in 4 ml of the same buffer, before being lysed by two passages through a cold French pressure cell at 18,000 lb/in^2. Unbroken cells and cell debris were removed by centrifugation at 15,000 x *g*, and the cell-free supernatant was applied to a nickel-nitrilotriacetic acid (Ni-NTA) affinity column (Qiagen, Valencia, CA). Buffer D was used to wash the resin until the A_{280} reached the baseline. Proteins were then washed with buffer E (buffer A with 30 mM imidazole) until the A_{280} reached the baseline, and the *Cc*BisA-His$_6$ recombinant protein was finally eluted with buffer F (buffer A with 250 mM imidazole). Both CcBisA and *Cc*BisA-His$_6$ proteins were dialyzed against Britton-Robinson buffer, pH 6.0 and stored in the dark at 4°C for subsequent application. The respective purity of each purified

protein was estimated by sodium dodecyl sulfate 12.5% polyacrylamide (SDS-PAGE) gel electrophoresis, and their respective concentration was determined with the BCA protein assay kit (Thermo Fisher Pierce, Rockford, IL, USA).

Disk Inhibition Assays. Briefly, WT (strains 13826 and 51562) cells and isogenic Δ*bisA* mutant cells were grown on BA plates for 24 h before being resuspended to a final A_{600} of 2 in sterile PBS buffer. Then 0.2 ml of cells were homogenously spread on top of 25-ml standardized BA plates (3–5 replicates per strain). A sterile paper disk (7.5 mm diameter) was placed in the center of each plate, and 10 μL of undiluted NaOCl (Sigma, 1.4 M, with NaClO ε_{292}=350 $M^{-1}cm^{-1}$) or 10 μL of 100 mM methyl viologen was added onto the disk. Cells were allowed to grow for 48 h, and the diameter of the inhibition zone was measured. Results shown are means and standard deviations from three to five replicates.
